# Digital Health Technologies Enabling Partnerships in Chronic Care Management: Scoping Review

**DOI:** 10.2196/38980

**Published:** 2022-08-01

**Authors:** Carolina Wannheden, Matilda Åberg-Wennerholm, Marie Dahlberg, Åsa Revenäs, Sara Tolf, Elena Eftimovska, Mats Brommels

**Affiliations:** 1 Medical Management Centre Department of Learning, Informatics, Management and Ethics Karolinska Institutet Stockholm Sweden; 2 Division of Physiotherapy School of Health Care and Social Welfare Mälardalen University Västerås Sweden; 3 Center for Clinical Research County of Västmanland Uppsala University Västerås Sweden

**Keywords:** participatory health, digital health, eHealth, collaborative care, participatory health informatics, cocare, partnership care management, chronic disease, long-term conditions, mobile phone, scoping review

## Abstract

**Background:**

An increasing number of patients expect and want to play a greater role in their treatment and care decisions. This emphasizes the need to adopt collaborative health care practices, which implies collaboration among interprofessional health care teams and patients, their families, caregivers, and communities. In recent years, digital health technologies that support self-care and collaboration between the community and health care providers (ie, participatory health technologies) have received increasing attention. However, knowledge regarding the features of such technologies that support effective patient-professional partnerships is still limited.

**Objective:**

This study aimed to map and assess published studies on participatory health technologies intended to support partnerships among patients, caregivers, and health care professionals in chronic care, focusing specifically on identifying the main features of these technologies.

**Methods:**

A scoping review covering scientific publications in English between January 2008 and December 2020 was performed. We searched PubMed and Web of Science databases. Peer-reviewed qualitative, quantitative, and mixed methods studies that evaluated digital health technologies for patient-professional partnerships in chronic care settings were included. The data were charted and analyzed thematically. The PRISMA-ScR (Preferred Reporting Items for Systematic Reviews and Meta-Analyses extension for Scoping Reviews) checklist was used.

**Results:**

This review included 32 studies, reported in 34 papers. The topic of participatory health technologies experienced a slightly increasing trend across publication years, with most papers originating from the United States and Norway. Diabetes and cardiovascular diseases were the most common conditions addressed. Of the 32 studies, 12 (38%) evaluated the influence of participatory health technologies on partnerships, mostly with positive outcomes, although we also identified how partnership relationships and the nature of collaborative work could be challenged when the roles and expectations between users were unclear. Six common features of participatory health technologies were identified: patient-professional communication, self-monitoring, tailored self-care support, self-care education, care planning, and community forums for peer-to-peer interactions.

**Conclusions:**

Our findings emphasize the importance of clarifying mutual expectations and carefully considering the implications that the introduction of participatory health technologies may have on the work of patients and health care professionals, both individually and in collaboration. A knowledge gap remains regarding the use of participatory health technologies to effectively support patient-professional partnerships in chronic care management.

## Introduction

### Chronic Care Model

Changes in demographics, disease panorama, and medical technology enabling early diagnoses and effective treatments have led to chronic diseases dominating the disease burden, accounting for alarming increases in health care use and costs [1]. Thirty years ago, Wagner et al [2,3] proposed the chronic care model (CCM), which called for a system of care that goes beyond health care provision by mobilizing supporting resources in the community. The model foresaw that the design and organization of service delivery would need to be adjusted to what was required and made possible by viewing chronic care as a system. Clinical information systems have been identified as important assets, particularly for decision support. In addition, self-management support for *informed and activated patients* has been emphasized [3]. Over the years, the CCM has inspired much of the development of chronic care management, as individuals with chronic conditions need services and support from several providers. Research has shown that more patients expect and want to play greater roles in decisions about their treatment and care and perform self-care more effectively [4-6]. Nevertheless, the transformation of health care practices into effective chronic care systems remains challenging [7].

### Collaborative Care Partnerships

The reference to *informed and activated patients* indicates that care arrangements according to the CCM are professionally driven, with health care professionals providing information and guidance to patients [3]. In contrast, patient-centered and person-centered care initiatives, launched at the start of the new millennium, highlighted the need to place patients at the center of their care and to make space for patient preferences in care planning, accomplished through a shared decision-making process [8]. This shift in care philosophy undoubtedly paved the way for patients with chronic conditions to be active rather than activated, which matched the aims of empowering patients and promoting equality in the patient-provider relationship. These movements emphasize the need to adopt collaborative health care practices, which implies collaboration between an interprofessional health care team and patients, their families, caregivers, and communities [9].

Central to this form of collaboration is the acknowledgment of patients as experts in their “experience, feelings, fears, hopes, and desires” [10]. Patient participation in co-design and *slow coproduction* helps strengthen their voices in the design of care services and can lead to improved patient experiences [11]. There is also evidence that when patients express what is important to them and have active roles in designing care, outcomes, including clinical outcomes, will improve [12]. In addition, several randomized controlled trials have shown that engaging patients in symptom monitoring, usually by applying digital technologies, has a positive effect on patient outcomes [13,14]. Symptoms are important not only to alert and guide the diagnostic workup but also to measure treatment effects (especially in severe illnesses) [15]. As patient-professional partnerships based on mutual respect for professional and experiential knowledge can strengthen patients in their self-management and shared decision-making with health care professionals, ultimately leading to improved clinical outcomes, it is worthwhile to study how such a collaborative care partnership can be enhanced.

### Participatory Health Technologies

Digital health technologies delivered in real time and in real-world settings offer opportunities to support such partnerships. Participatory health informatics, which emerged as a field around 2008, concerns the use of “information technology as provided through the web, smartphones, or wearables to increase participation of individuals in their care process, and to enable them in self-care and decision-making” [16]. For example, web-based social health networks such as PatientsLikeMe [17] have become powerful tools for patients to share their experiences and learn from each other. Technologies for community support marked the beginning of participatory health technologies, and interest has been increasing in technologies supporting self-care and patient-professional partnerships, which was the focus of this study. In particular, the use of text-based patient-professional communication tools has increased over the past decade [18], supporting self-management and contributing to increased patient participation [19]. Although the use of mobile health apps generally has a positive influence on patient-professional relationships, health care professionals may still be reluctant to use them [20]. In addition, despite these apps’ potential to improve health care delivery to people with chronic conditions, their effects on health outcomes have been found to be inconsistent [19,21]. Thus, there is a need to gain more knowledge about the mechanisms that contribute to effective patient-professional partnerships. Therefore, this study aimed to map and assess published studies on participatory health technologies intended to support partnerships between patients, caregivers, and health care professionals in chronic care, focusing specifically on identifying the main features of these technologies.

## Methods

### Study Design

A scoping review was considered relevant as our aim was to examine the size, scope, and nature of the available literature on our phenomenon of interest and summarize existing research findings [22]. The review was performed in 5 stages, guided by the Arksey and O’Malley framework [23,24]. A review protocol (available on request) was developed beforehand and continuously updated to ensure consistency and reproducibility. The review team covered multiple areas of relevant expertise, including health informatics, health services research, medical technology management, and medicine. A list of experts in the domains of health care, patient self-care, and digital health was established to be contacted if expert advice was needed. For example, we sought and obtained input on the practical relevance of our research questions.

### Stage 1: Identifying the Research Question

The scoping review question was specified by considering the population, intervention, comparison, and outcome [25]. The population of interest was broadly limited to people living with chronic illnesses; the intervention of interest was specified in detail, focusing on digital health technologies that enable partnerships between patients, caregivers, and health care professionals (ie, participatory health technologies). No comparison method was specified, and we aimed to identify all types of outcomes explored in previous studies. We posed the following overarching research question: what is known from the existing literature about participatory health technologies that intend to support partnerships between patients, caregivers, and health care professionals in chronic care? More specific research questions were posed in line with our aim:

- The context of use: At which levels of care are the participatory health technologies used? For which types of chronic conditions are participatory health technologies used? Who are the users of participatory health technologies?
- Evaluation: What study designs are used and what outcomes are measured and reported?
- Features supporting partnerships: What are the main features of participatory health technologies? How do the different features influence partnerships?

### Stage 2: Identifying Relevant Studies

The search strategy was developed in consultation with the Karolinska Institutet University Library, following the Peer Review of Electronic Search Strategies guidelines [26]. Search terms were designed to capture papers related to three key concepts: (1) digital health technologies, (2) partnerships between patients, caregivers, and health care professionals, and (3) chronic care management. Searches were performed using the bibliographic databases PubMed and Web of Science, which were considered most relevant in relation to our aim. First, we identified synonymous terms for each key concept and combined them into a search phrase using the Boolean operator OR. We also identified and used relevant Medical Subject Heading terms in PubMed. We then combined the search phrases for the 3 concepts using the Boolean operator AND. The exact search phrases for the 2 databases are presented in Multimedia Appendix 1. Our searches were performed on November 21, 2017, and updated on December 14, 2020. The search results were filtered by language and time span, covering papers in English and Swedish published between January 2008 and December 14, 2020.

### Stage 3: Study Selection

The inclusion and exclusion criteria are listed in Table 1. The period of inclusion, from 2008 to 2020, was motivated by the emergence of the term *participatory health informatics* in 2008 [16]. Screening was performed using the open-source platform Rayyan [27]. We specified the labels to be used as reasons for exclusion in the screening process if the inclusion criteria were not met. At the beginning of the screening process, the inclusion criteria were piloted and refined in several iterations until a consensus was reached among all authors. The first screening was performed in late 2017 and early 2018 by EE and MÅW with support from MD, ST, ÅR, and CW; titles and abstracts for each study were screened by at least two of these researchers (blinded). Conflicts were resolved through discussion and, if necessary, by involving the research team. The second screening, following an updated search, was performed in early 2021 by CW and MB who both screened all titles and abstracts and resolved conflicts through discussion. They also screened the reference lists of the included studies to identify additional relevant publications.

**Table 1:** Inclusion and exclusion criteria.

Criteria		Inclusion	Exclusion
Type of studies		Qualitative, quantitative, and mixed methods studies on the phenomenon published in peer-reviewed journals	Letters, commentaries, editorials, conference abstracts, doctoral theses, or any type of review
Period		January 1, 2008, until December 14, 2020	Before January 1, 2008, and after December 14, 2020
Language		English and Swedish	All other languages
Type of participants		Patients with chronic conditions, defined as a health condition that lasts at least 3 months	Patients who do not have chronic conditions
**Phenomenon of interest**		Studies that meet all 3 criteria listed below	Studies that do not meet all the 3 criteria listed below
	Digital health technology	A digital health technology is defined as software intended for use for *preventive, promotive, curative, rehabilitative, assistive, or palliative care*; this includes categories such as eHealth or mobile health, wearable devices, and telehealth services; the digital health technology should enable processing and exchange of health information between end users using the *internet*	Nondigital services or digital services not specifically intended for medical use; for example, WhatsApp, email, telephone, and SMS text messages are technologies that are not primarily intended for the abovementioned purposes and were thus excluded
	Partnership	The digital health technology intends to support collaboration and enables interaction between at least two types of users: patients or caregivers, and health care professionals or allied professionals (eg, pharmacists)	Digital health services for peer-to-peer collaboration between patients or caregivers only, or tools for team collaboration among staff, without patient or caregiver involvement, were excluded; tools that only intended to support self-care or treatment adherence were also excluded
	Evaluation	Evaluation results testing the digital health technology in chronic care need to be available	Studies that merely describe the design and development of digital health technologies; evaluation that has not been performed in a real-world setting (eg, heuristic evaluation by experts)

### Stage 4: Charting the Data

A data extraction sheet was developed containing bibliometric variables (author, country based on corresponding author affiliation, title, year, and journal), descriptive study variables (study aim, study design, and sample size), and variables based on the research questions (chronic condition, level of care, name and description of the participatory health technology, outcome measures, and evaluation results). All authors were involved in testing and refining the data extraction sheet with a selection of papers. A total of 2 authors per paper extracted and compared these to calibrate the variable definitions and our shared understanding thereof. Thereafter, CW and MB performed the remaining extractions for all the papers.

### Stage 5: Collating, Summarizing, and Reporting Results

Charted data were condensed and grouped into categories that enabled the classification of the studies based on their study aims, study designs, chronic conditions, outcome measures, and evaluation results. The charted text describing the participatory health technology features and their influences on partnerships was extensive and was, therefore, analyzed separately using a qualitative content analysis process [28]. The charted text was abstracted through text condensation and categorization, which was performed by CW and discussed with MB. Meaning units were identified and coded inductively using a Microsoft Excel spreadsheet. Thereafter, all codes were transferred to the open source FreeMind mind-mapping software [29], where they were grouped into categories and subcategories. After categorizing all charted data, we used the statistical software R (R Foundation for Statistical Computing) [30] to explore descriptive statistics and the ggplot2 package [31] to produce visualizations. We first present a descriptive numerical summary of the included papers and then present an inductive categorization of the main participatory health technology features, supported by illustrative examples. The PRISMA-ScR (Preferred Reporting Items for Systematic Reviews and Meta-Analyses extension for Scoping Reviews) checklist for scoping reviews was used for reporting.

## Results

### Study Selection

**Figure 1:**
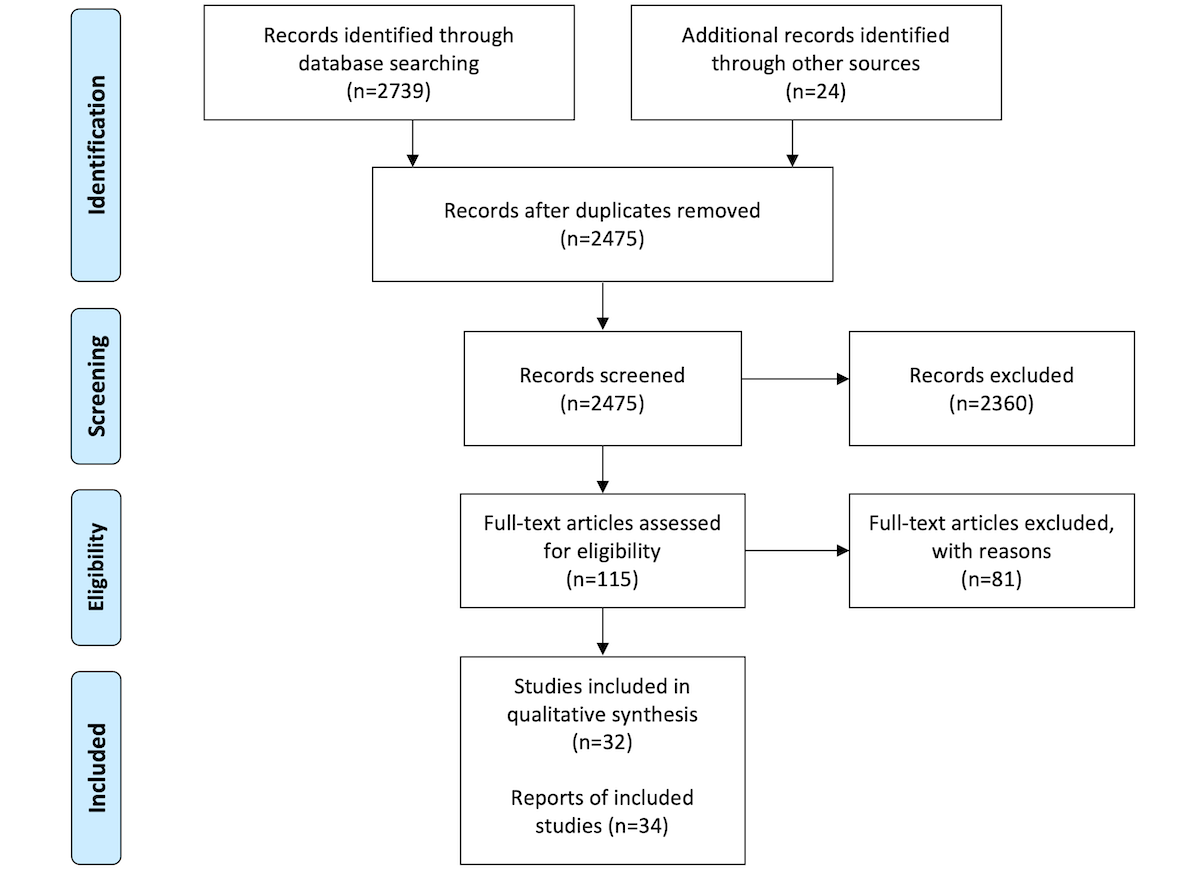
PRISMA (Preferred Reporting Items for Systematic Reviews and Meta-Analyses) flow chart.

Database and manual searches yielded 2763 records (Figure 1); after removing duplicates, the titles and abstracts of 2475 (89.58%) records were screened, and 2360 (85.41%) records that did not meet the inclusion criteria were removed. We read the full texts of 115 papers and excluded 81 (70.4%) for the following reasons: not partnerships (n=67, 58.3%), not digital health technologies (n=11, 9.6%), not used in care (n=1, 0.9%), not evaluated (n=1, 0.9%), and not original research (n=1, 0.9%). The remaining 34 papers, reporting on 32 studies of 30 participatory health technologies, were included in the qualitative synthesis. The characteristics of individual papers are presented in Multimedia Appendix 2 [32-65]. In our presentation of study characteristics, data were consolidated from publications reporting on the e-BP study [33,34] and the MyCyFAPP study [43,44].

### Study Characteristics

#### Publication Details

**Figure 2:**
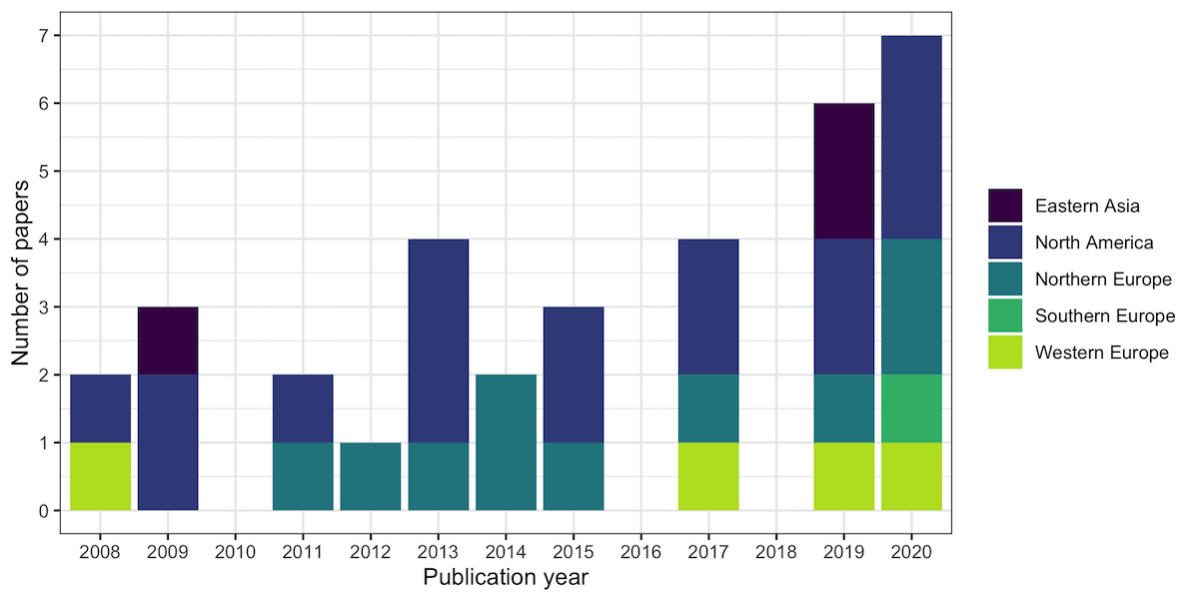
Number of included papers by year and world region.

The topic of participatory health technologies experienced a slightly increasing trend across the publication years, with most papers being published in the past 2 years of the review period (Figure 2). The papers originated from the United States (15/34, 44%), Norway (7/34, 21%), and China (2/34, 6%), and a single (1/34, 3%) paper each from the following countries: Canada, Denmark, Sweden, the United Kingdom, the Netherlands, Germany, Austria, Switzerland, Spain, and South Korea. They were published in 21 journals, most commonly the *Journal of Medical Internet Research* (5/34, 15%), *JMIR mHealth and uHealth* (5/34, 15%), *Telemedicine and eHealth* (3/34, 9%), and the *International Journal of Medical Informatics* (3/34, 9%).

#### Context of Use

The studies were conducted in primary care (18/32, 56%), secondary care (18/32, 56%), and tertiary care (7/32, 22%; Table 2). Approximately one-third (10/32, 31%) of the studies were set across levels of care, and some (3/32, 9%) additionally involved home care, social services, or school health services. Most studies addressed diabetes (8/32, 24%), followed by cardiovascular disease (6/32, 19%). The participatory health technologies were deployed almost exclusively as web applications or websites for health care professionals, whereas mobile deployment was common for patient users (14/32, 44%). In some studies, participatory health technologies were integrated into electronic health record systems or personal health records [32-38]. In addition to interactions between patients and health care professionals, participatory health technologies supported collaboration with allied professionals [33,34,36,39] or technical staff [40,41]. Caregivers were identified as users in some studies in which the patients were children [37,42-45], cognitively impaired [46], or in palliative care [47]. We identified 3% (1/32) of studies in which patients who did not meet these criteria had the option to invite their families and friends to be users of participatory health technology [48].

**Table 2:** Context of use.

Characteristic			References
**Level of care**			
	Primary care	[33-40,42,46,48-56]	
	Secondary care	[32,39,40,42-45,47,49,52,55-63]	
	Tertiary care	[39,41,43,44,58,60,64,65]	
**Type of chronic condition**			
	Metabolic (diabetes)	[35,38,51,53,55,57,64,65]	
	Cardiovascular	[32-34,36,39,48,61]	
	Autoimmune	[45,49,58-60]	
	Pulmonary	[37,48,54]	
	Cancer	[47,62,63]	
	Genetic	[43-45]	
	Immunodeficiency	[40,41]	
	Psychiatric	[42]	
	Neurodegenerative	[46]	
	Unspecified	[50,52]	

#### Evaluation

Of the 32 studies, 13 (41%) were effect studies of participatory health technology use in clinical practice, 8 (25%) were feasibility studies, 7 (22%) explored user experiences, and 4 (13%) reported on the design and implementation of participatory health technologies (Table 3). The study designs included randomized clinical trials (11/32, 34%), quantitative evaluations (8/32, 25%), qualitative evaluations (7/32, 22%), and mixed methods evaluations (6/32, 19%). Of the 32 studies, the sample size was as high as 50 in 11 (34%) studies, 50 to 200 in 14 (44%) studies, and >200 in 7 (22%) studies. The studies evaluated the effects of participatory health technologies on clinical outcomes, including health, well-being, quality of life (17/32, 53%), user experiences (12/32, 38%), and self-management (7/32, 22%). Approximately one-third (12/32, 38%) of studies evaluated effects on partnerships by describing the content, experiences, and nature of collaboration [39,48,51,56]; the distribution of tasks and responsibilities [42]; patient-professional relationships [51,54]; engagements of patients and family caregivers [41,45,46]; and the perceived quality of collaborations [45,46]. Other effects that were evaluated included access to care and waiting times [49], continuity of care [47], and health care costs [34,52]. Most studies reported positive outcomes (22/32, 69%), although they were minor or temporary in some cases [35,62]. A few studies reported mixed results (5/32, 15%) or no change (3/32, 9%).

**Table 3:** Study designs and outcomes.

Characteristic			References
**Study aim**			
	Design and implementation	[39,40,61,64]	
	User experiences	[42,46,48,50,51,56,59]	
	Feasibility	[32,37,45,47,53-55,60]	
	Effects	[33-36,38,41,43,44,49,52,57,58,62,63,65]	
**Study design**			
	Randomized controlled trial	[33-35,37,38,47,57,58,62,63,65]	
	Quantitative	[36,41,43,45,52,53,55,60,64]	
	Qualitative	[39,40,42,46,48,50,51]	
	Mixed methods	[32,44,54,56,59,61]	
**Sample size**			
	≤50	[39,42,46,48,50,51,54,55,60,61,64]	
	51-100	[32,37,38,45,47,56,57]	
	101-150	[36,41,53]	
	151-200	[40,43,44,58,63]	
	>200	[33-35,49,52,59,62,65]	
**Outcome variables**			
	Clinical outcomes	[32-38,41,43,44,47,55-58,62-65]	
	Partnership	[37,39-42,44-46,48,51,54,56]	
	Self-management	[32,36,44,45,57,62,63]	
	User experiences	[32,37,44-46,48,50,56,57,59,60,64]	
**Outcomes**			
	Positive outcomes	[32-37,39,41,43-46,48,49,53-55,57,59,60,62-65]	
	Mixed results	[42,47,50,51,56]	
	No change	[38,52,58]	
	N/A^a^	[40,61]	

^a^N/A: not applicable.

### Qualitative Synthesis of Participatory Health Technology Features and Their Influences on Partnerships

#### Overview of Features

**Figure 3:**
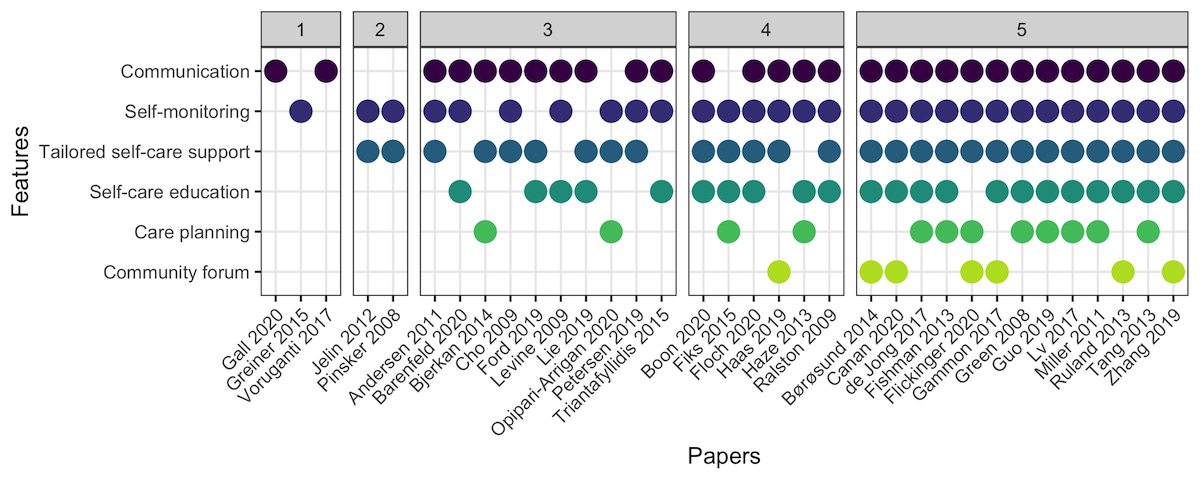
Illustration of identified participatory health technology features in each of the included papers, grouped by the total number of features (ranging from 1 to 5) [32-65].

We identified six main participatory health technology features for enabling partnerships between patients and health care professionals: (1) communication, (2) self-monitoring, (3) tailored self-care support, (4) self-care education, (5) care planning, and (6) community forum (Table 4). Most studies described a combination of these features, often involving the first 3 (Figure 3 [32-65]). In the following sections, we describe the 6 features and their influences on patient-professional partnerships that were discussed in the studies.

**Table 4:** Thematic analysis of participatory health technology features.

Themes (features)			References
**Communication**			
	Asynchronous message exchange	[32-36,38,40-44,46-49,52-57,60-63]	
	Audio or video communication	[32,39,48,52]	
	Unspecified	[65]	
**Self-monitoring**			
	Self-measurements of health parameters	[33-36,52,53,57,59,61,65]	
	Self-assessment of symptoms or problems	[32,37,39,43-45,50,58,62,63]	
	Self-reported health status or activity	[32,35,38,40,41,44,45,48,50,54-56,59-61,64,65]	
	Self-reported medication adherence or side effects	[32,35,38,40,44,64]	
	Diary for personal notes	[48,55,63]	
**Tailored self-care support**			
	Personalized goals	[34-37,44,51,55,64]	
	Medication management	[32-34,36,39,41,49,56,64,65]	
	Individual feedback	[35,50,51,55,64]	
	Tailored recommendations	[33-35,37,43-45,49,52,57,62,63]	
	Alerts and reminders or prompts	[32,36-38,41,42,45,52,57,58,60,64]	
**Self-care education**			
	Educational material integrated in participatory health technology	[32,34-37,41,43,44,49,51,53,54,56,60-62,65]	
	Links to external sources	[32,33,38,48,58,62,63]	
**Care planning**			
	Access to a personal care plan	[32,34-37,40,42,54]	
	Appointments and previsit planning	[33,45,58,60]	
**Community forum**			
	Anonymous contributions	[40,41,55,56,62,63]	
	Health care professional monitored	[55,56,63]	
	Unspecified	[65]	

#### Communication

Support for patient-professional communication was a central participatory health technology feature described in 84% (27/32) of studies. Most commonly, communication was facilitated through asynchronous text-based information exchanges between users; however, some studies also implemented audio- or video-based communication. In some studies, team-based communication between multiple users (including patients, caregivers, care team members, and allied health professionals) was also supported, enabling patients and caregivers to communicate with multiple care team members and care team members to interact with each other [46,47,49]. This communication feature contributed to rapport building [40] and improved patient-professional relationships among young and adult patients [44,54,60]. In a study of teenagers living with asthma, health care professionals reported that written communication could lead to more honest and elaborate responses among patients who may be less talkative in face-to-face encounters [54]. Meanwhile, a study that evaluated e-consultations for diabetes self-management support found that asynchronous communication could make patient-nurse relationships more fragile because of the risk of misunderstandings, suggesting that the best option may be a combination of written and face-to-face interactions [51]. Most studies did not describe any constraints in content, time, or word limits for message exchanges. Although one of the studies reported that health care professionals did not experience answering messages as too time consuming [63], other studies reported that tighter communication and follow-up of patients led to greater workloads for health care professionals between consultations [44] and could blur the boundaries between their private and work lives [56]. Various engagements with participatory health technologies among health care professionals and patients led to frustration when expectations were not met, for example, when messages were not answered [42,56].

#### Self-monitoring

Self-monitoring was also a central feature found in nearly all studies (27/32, 84%). It comprised the use of self-measurement devices to register and report health data, such as blood pressures [33,34,36,61,64], blood glucose levels [35,53,57,64,65], or physical activity [36,52]; self-assessments of symptoms or problems; self-reported health statuses or activities; self-reported medication adherence or side effects; and diaries for personal notes. When health parameters were not measured using external devices, self-monitoring was mostly facilitated through structured data input based on predefined forms. Several benefits regarding patient-provider partnerships were identified: increased patient motivation [50,59], higher perceptions of being recognized and respected by health care professionals [48], and more efficient consultations because of less time being spent on collecting and explaining data [44]. By providing contextual information and their own interpretations of self-monitored data, patients could participate as diagnostic agents in clinical assessments [39].

#### Tailored Self-care Support

The features for providing tailored self-care support were identified in 78% (25/32) of studies. This entailed support for setting and monitoring the progression toward personalized goals, medication management (eg, personal medication lists and managing refills), individual feedback, tailored recommendations, and alerts and reminders. Health care professionals provided individual feedback to patients on the basis of clinical variables or reported self-assessments and reflections [35,50,51,55,64]. In addition to feedback, tailored recommendations were often provided regarding therapy adjustments, symptom management, self-management activities, self-monitoring, and topics to discuss with clinicians. Recommendations were either automatically generated based on patients’ reported data [37,43-45,52,62,63] or individually tailored by health care professionals [34,35,49,57]. The provision of feedback contributed to the development of good relationships and made patients feel understood and addressed, although feedback could also be experienced as challenging for patients [50]. Motivational messages could be both appreciated and experienced as annoying [44]. Alerts were provided to draw attention to patients and health care professionals, generally based on predefined threshold values for clinical parameters [32,36,45,60,64]. In some cases, alerts were also used to inform health care professionals about patients’ activities or engagement with participatory health technologies [42,52]. Reminders were used to support medication adherence [41], prompt patients to upload self-monitoring data [37,57,58,64], or remind them of clinical examinations or appointments [38].

#### Self-care Education

Features for providing self-care education were described in almost two-thirds of the studies (20/32, 62%). Educational material was integrated into the participatory health technologies or provided through links to external sources and often covered both disease-specific information and lifestyle topics, such as nutrition, health and wellness, or smoking cessation [41,43,44,60]. Where educational material was embedded in participatory health technology, contents could be adapted specifically to the target group. For example, in a study of patients with diabetes [53], educational material was adapted to be culturally appropriate to the target group of native communities. Self-care education was sometimes delivered through video clips [35,37,54,61] or could include a toolbox of resources, such as recommended activities, good-to-know texts, and workbooks [56]. Several studies reported improvements in self-management knowledge and self-efficacy [32,43,44,60].

#### Care Planning

Approximately one-third (11/32, 34%) of the studies described features for participatory health technology–supported care planning. This involved access to planned activities or personal health plans and support for scheduling appointments or planning care visits. In preparation for care visits, patients had opportunities to identify goals, questions, or problems to discuss with their clinicians and provide information about their disease activity by filling in structured data forms [45,58,60]. A study of pediatric patients [45] found that visits and collaborations improved through this preparation. Another study identified shifts in roles and sometimes power transitions from health care professionals to patients and caregivers as they took more responsibility for care planning [42].

#### Community Forum

Web-based community forums for peer-to-peer interactions with other patients were provided in some studies (7/32, 22%). This functionality appeared in studies published in 2013 or later and only in participatory health technologies that had several other features as well. In most cases (5/7, 71%), the community forums enabled patients to write questions and comments anonymously to protect their integrity. In 43% (3/7) of studies, community forums were monitored by health care professionals who could contribute with answers to posted questions. In a community mental care setting, it was reported that peer support initially established through an anonymous community forum could develop into friendships when combined with café gatherings where service users could meet in real life [56]. Several studies found that patients would visit the web-based community forums to read others’ posts more often than to post something themselves [41,56,62].

## Discussion

### Principal Findings

This scoping review identified and described the characteristics of participatory health technologies supporting patient-professional partnerships in chronic care management evaluated in 32 studies and published in 34 papers. These papers originated almost exclusively from North America and Europe and were published in a variety of journals, mainly in the fields of biomedical informatics or information science but also in the fields of health services research, medicine, and nursing. This reflects the multidisciplinary nature of participatory health informatics in chronic care management. The slight increase in the publication trends may suggest an increasing interest in digital services for participatory medicine in recent years. Notably, the included papers represented high variation in terms of the chronic conditions addressed, the levels of care where the participatory health technologies were used, the study designs, and the sample sizes. Nevertheless, 6 common participatory health technology features could be identified. Most participatory health technologies had features to support patient-professional communication, self-monitoring, and tailored self-care support. More than half of the studies described self-care education features, and approximately one-third discussed features to support care planning. In more recently published studies, the facilitation of peer support through web-based community forums emerged as a new feature. The engagement of caregivers as participatory health technology users was also more common in recent studies, possibly indicating a shift from focusing merely on the patient-professional dyad to a system view of collaborative care, acknowledging the involvement of more stakeholders. Most studies reported positive outcomes, although there were mixed results, highlighting the importance of tailoring participatory health technology implementation and use to individuals’ needs and preferences.

### Comparison With Prior Work

Our thematic analysis focused on identifying common participatory health technology features and describing the identified influences on patient-professional partnerships. In the following sections, we discuss 3 observations made when interpreting our findings, namely, how participatory health technologies influence roles and relationships, the changing nature of chronic care work, and a shift from intermediation to apomediation.

#### Changing Roles and Relationships

Overall, our findings are in line with previous studies indicating that the use of eHealth interventions can positively influence patient-professional communication and relationships [20] and also challenge these because of undefined or changed roles [66]. As a previous review has shown [20], the positive influences of participatory health technologies on patient-professional relationships depend on participating actors who meet the expectations and rules of minimal engagement. This was clearly seen in some studies in mental care [42,56], where variation in patient or professional engagement with participatory health technologies could lead to either enhanced or challenged relationships. One of these studies highlighted that health care professionals may need to communicate their personal boundaries to patients; for example, they would only check messages on certain weekdays [56]. However, these kinds of social interaction norms have rarely been made explicit in studies where asynchronous interactions were not structured or constrained, which could lead to a blurring of the boundaries that define the contents, extents, and times of interactions [67,68]. The ethical implications of digital patient-professional communication can be complex and require organizational guidelines to promote good practices in the use of digital communication [67]. Role uncertainty may affect both staff and patients [69], suggesting that the introduction of participatory health technology features that enable unlimited asynchronous interactions or task shifting (eg, the patient takes on tasks traditionally performed by health care professionals) should also involve mutual agreements on the distribution of tasks, roles, and responsibilities between patients and professionals.

#### Changing Nature of Collaborative Chronic Care Work

In addition to communication support, the most common participatory health technology features we identified were self-monitoring and tailored self-care support, which is comparable with the results of a recent scoping review focusing on the features of web portals for telerehabilitation [70]. These 3 features were often combined, and they have the potential to profoundly influence the nature of collaborative work among patients, caregivers, and health care professionals. Through self-monitoring and self-care, patients take over tasks that were previously performed by health care staff (eg, measuring of vital parameters) or not performed at all (eg, continuous collection of health parameters between consultations). As described in one of the papers in our review [44], patients’ self-monitoring also influenced the work of health care professionals in several ways. Consultations could become more efficient as data were collected in real time and made available to both patients and health care professionals before consultations. In contrast, health care professionals had to spend more time between visits responding to questions or providing feedback to their patients (ie, communication and tailored self-care support). This indicates that health care professionals’ workloads may increase in some areas but decrease in others, with implications for how their work is organized. Workloads, workflow disruptions, and alignment with clinical processes are among the most common barriers to the adoption of eHealth services [66]. Another study found that when patients’ transmission of data replaced physical meetings, the patients could become passively disengaged, resulting in poorer collaborations [39]. Enabling patients to provide contextual information in addition to automated self-measurements contributed to reintroducing them as collaborative partners in diagnostic interpretation; however, the authors questioned whether this could really be labeled as collaboration or merely the transmission of more data. This study clearly problematized the potential issues when self-monitoring merely replaced previous collaborative work. When self-measured data are not interpreted in collaboration with the patient, the partnership may be harmed rather than improved.

Features for care planning provided another example in which the nature of collaborative work could change. For example, care planning enabled patients to influence the agenda for care visits by communicating their personal goals and the questions they wanted to address. One of the studies described a power transition, as patients took more responsibility for their care plans [42]. Altogether, these findings emphasize that the potential implications of participatory health technologies on the nature of collaborative work need to be carefully considered when introducing eHealth services that influence the work of patients and health care professionals in chronic care management.

#### Moving From Intermediation to Apomediation

With the rise of web-based technologies, referred to as Web 2.0, and similarly, Medicine 2.0, the terms intermediation, disintermediation, and apomediation were introduced [71,72]. Intermediation refers to the selection and delivery of “relevant” health information by an intermediary (eg, health care professionals or a web portal vetted by experts). For example, self-care education and self-care support features that were quite common in this review may be understood as methods of intermediation. By providing patients with relevant self-care information, health care professionals can shift away from the paternalistic model of physician-patient relationships to an interpretive model, where they take on roles as counselors or advisers in individuals’ self-care [73]. The provision of self-care education was associated with increased knowledge and self-efficacy, which are resources that individuals can draw on to build their capacities for self-management [74]. It has been suggested that the more knowledgeable and self-efficacious patients become, the less they want to rely on experts (ie, disintermediation), preferring guidance from peers who “stand by” rather than “in between” patients and the knowledge they seek (ie, apomediation) [71]. An example of apomediation is web-based social health networks, which have been integrated as components in the eHealth-enhanced CCM [75]. Although web-based communities marked the beginning of participatory health informatics [16], the integration of social networking features in participatory health technologies intended for patient-professional interactions emerged as a new trend in this study. Our results illustrate that the 3 different types of participatory health technologies that have been previously distinguished (ie, Web 2.0, self-care support, and tools supporting health care provision) are being increasingly combined in multimodal services. This suggests that participatory health technologies may indeed enable a shift toward a more collaborative and networked approach to participatory medicine beyond the patient-professional dyad. We have identified several features to support partnerships in chronic care management; however, the processes of how patient knowledge is shaped and integrated in shared decision-making are still poorly characterized [76]. Future research may reveal how knowledge from web-based health communities, patients, caregivers, and health care professionals can be effectively combined to support patients in their individual self-care and drive quality improvement and collective organizational learning.

### Strengths and Limitations

This scoping review has several strengths, including the inclusion of all types of study designs to obtain findings assessed using different methods, a screening method involving multiple researchers, and a qualitative synthesis contributing to new knowledge. The included studies covered various chronic conditions, clinical settings, and study designs. Our search strategy limited the review to papers published in English and Swedish between 2008 and 2020, implying that we may have missed important studies published earlier and in different languages. Furthermore, the inclusion criteria restricted the studies to those that reported the use of software specifically intended for clinical use (ie, excluding the use of email, SMS text messages, or nondigitally supported means of partnership) and had been evaluated in clinical practice. Nevertheless, our findings add new knowledge that contributes to describing the scope and nature of participatory health technology features to support patient-professional partnerships. Only 38% (12/32) of studies evaluated the effects on partnerships, which suggests that a knowledge gap remains regarding the influence of participatory health technologies on the nature of partnerships and how to support collaborative health care practices effectively. As most studies reported positive results, there may also be a publication bias, given that studies of failed eHealth interventions are published less frequently [77].

### Conclusions

This scoping review identified participatory health technologies evaluated in studies intending to support partnerships between patients and caregivers and health care professionals in chronic care and qualitatively analyzed the main features of these technologies. A total of 6 common features were identified: patient-professional communication, self-monitoring, tailored self-care support, self-care education, care planning support, and community forums for peer-to-peer interactions. The integration of social networking features for community support in health technologies intended for patient-professional interactions is an emerging trend, which suggests a shift toward a more collaborative and networked approach to participatory medicine beyond the patient-professional dyad. The studies in this review mainly reported positive outcomes; however, we also identified how partnership relationships and the nature of collaborative work could be challenged when roles and expectations between users were unclear. This emphasizes the importance of clarifying mutual expectations and carefully considering the implications that the introduction of participatory health technologies may have on the work of patients and health care professionals, individually and in collaboration. Future research should further explore the mechanisms by which participatory health technologies contribute to the shaping and use of collaborative knowledge to benefit individual patients, patient populations, and organizational learning.

## Appendix

Multimedia Appendix 1 Search strings.

Multimedia Appendix 2 Characteristics of included papers.

## Abbreviations

CCM: chronic care model
PRISMA-ScR: Preferred Reporting Items for Systematic Reviews and Meta-Analyses extension for Scoping Reviews
