# Supplementary material for: Digital Health Technologies Enabling Partnerships in Chronic Care Management: Scoping Review
Source: J Med Internet Res. 2022 Aug 1;24(8):e38980. doi: 10.2196/38980 (PMC9379797; doi:10.2196/38980)
Supplement: Multimedia Appendix 1 [file jmir_v24i8e38980_app1.pdf]

This is a Multimedia Appendix to a full manuscript published in the J Med Internet Res. For full copyright and citation information see http://dx.doi.org/10.2196/38980

Multimedia Appendix 1: Search strings

Concept	PubMed search string	Web of Science search
Digital health technologies	(Telemedicine[MeSH Terms] OR m-health[tiab] OR mhealth[tiab] OR e-health[tiab] OR Ehealth[tiab] OR Internet[MeSH Terms] OR Medical Informatics[MeSH Terms] OR internet*[tiab] OR digital[tiab] OR smartphone*[tiab] OR Web[tiab] OR Computers, Handheld[MeSH Terms])	TS=(Telemedicine OR Medical Informatics OR internet* OR smartphone* OR web OR digital* OR m-health OR mhealth OR e-health OR ehealth)
Partnership between patients/ caregivers and healthcare professionals	(Professional-Patient Relations[MeSH Terms] OR Patient-Centered Care[MeSH Terms] OR person centered care[tiab] OR patient centered care[tiab] OR partnership[tiab] OR shared-decision*[tiab] OR participat* medicine[tiab] OR co-production[tiab] OR co-creation[tiab] OR e-patient[tiab] OR epatient[tiab])	TS=("Professional-Patient Relation" OR "Patient-Centered" OR shared-decision* OR co-production OR co-creation OR partnership OR "person centered" OR patient centered care OR participat* medicine OR e-patient OR epatient)
Chronic care management	(Chronic Disease[MeSH Terms] OR Long-Term Care[MeSH Terms] OR chronic-care[tiab] OR chronic condition*[tiab] OR long term condition*[tiab] OR self-management[tiab] OR self-care[MeSH Terms])	TS=("Chronic Disease" OR Long-Term care OR self-management OR self-care OR chronic* OR long term condition*)

Correspondence: Carolina Wannheden, carolina.wannheden@ki.se
