# Supplementary material for: Digital Health Technologies Enabling Partnerships in Chronic Care Management: Scoping Review
Source: J Med Internet Res. 2022 Aug 1;24(8):e38980. doi: 10.2196/38980 (PMC9379797; doi:10.2196/38980)
Supplement: Multimedia Appendix 2 [file jmir_v24i8e38980_app2.pdf]

This is a Multimedia Appendix to a full manuscript published in the J Med Internet Res. For full copyright and citation information see http://dx.doi.org/10.2196/38980

Multimedia appendix 2 – Paper characteristics

Description of participatory health technologies (PHTs) and identified features

First author, publication year	PHT description
**Andersen, 2011**	**myRecord**: Add-on web application that is used together with a telemonitoring system and supports the interpretation of the patient's general condition and the patient’s interpretation of their own condition and of data recorded by an implantable cardioverter-defibrillator.
**Barenfeld, 2020**	A smartphone application, featuring a secure web-based design with a dashboard including health assessment; personalized health plan; disease-specific education; and communication for follow-up evaluation.
**Bjerkan, 2014**	**SamPro**: A web-based tool that provides access to a patient's individual care plan, activity logs, SMS, and an internal email module.
**Boon, 2020**	**MyCyFAPP**: A mobile application with four main features: a diary where patients record planned meals; a symptoms diary; a link to the professional webtool with follow-up charts; and educational material about nutrition.
**Børøsund, 2014**	**WebChoice**: A web-based illness management support system to help cancer patients reduce their symptom distress, improve emotional wellbeing, and enhance self-efficacy. WebChoice includes: an assessment component for patient self-monitoring; an advice component providing illness self-management support; an information component; a communication component for sharing experiences with other patients; an electronic diary; and patient-provider communication.
**Canan, 2020**	**PositiveLinks (PL)**: A clinic-based mobile platform to increase engagement in care among people living with HIV. A patient mobile app contains the following features: medication reminders; self-rated mood, stress, and medication adherence; provider messaging; informational resources; and an anonymous community board that allows users to interact with one another.
**Cho, 2009**	**Diabetes Phone**: A mobile phone containing a device to measure capillary blood glucose on site and transmit blood glucose data to a web server automatically without manual input. The diabetes phone has the same functions as other mobile phones, including SMS and an alarm.
**de Jong, 2017**	**myIBDcoach**: A secure webpage and HTML application on a tablet or smartphone that includes monitoring modules, intensified monitoring modules, outpatient visit modules, e-learning modules, a personal care plan, communication, and an administrator page used by the health care provider.
**Fiks, 2015**	**MyAsthma**: An EHR-linked patient portal with decision support directed at families and clinicians, including the following features: identification of parents' concerns and goals for asthma treatment; monthly tracking of symptoms, medication side effects, and goal progress; educational content including videos; and access to the child's care plan.
**Fishman, 2013**	**e-BP**: A website that enables members to communicate with their providers through secured messaging, schedule appointments, refill prescriptions that are then mailed, and view most of the medical record including lab results. In addition to the website, a home blood pressure monitor and direct care supervision from a clinical pharmacist.
**Flickinger, 2020**	**PositiveLinks (PL)**: A smartphone application with secure patient–provider messaging, including access to appointments and laboratory data; daily queries related to medication adherence, mood, and stress; and a community message board for secure anonymous communication with other patients.
**Floch, 2020**	**MyCyFAPP**: A self-management app targeting parents of young children with CF and teenagers with CF and a professional web tool targeting healthcare professionals. Features: calculation of personalized enzyme dose; follow-up of food intake; food recording; access to recommended country-specific dishes; health diary for recording mood and GI symptoms; and an educational handbook about disease and treatment.

Correspondence: Carolina Wannheden, carolina.wannheden@ki.se

**Ford, 2019**	A web-based platform that enabled prompt receipt of dermatologist expertise and sharing of visit information among patients, primary care practitioners, and dermatologists. Features: sending of digital photos and clinical history; treatment recommendations; prescriptions; and patient educational materials.
**Gall, 2020**	**CareShare**: A collaborative communication system deployed as a cloud-based application that provides browser interfaces for mobile devices. Features: personal profile; interactions between residents and caregivers using "situation cards" with textual descriptions of observable cues; "heart openers" that provide conversational topics linked to media files (images, videos, music, texts); group chat with associated caregivers and significant others.
**Gammon, 2017**	**ReConnect**: A recovery-oriented eHealth portal consisting of (1) a toolbox of resources for articulating and working with recovery processes, such as: status/goals/activities relative to life domains (e.g., employment, social network, health); medications; network map; and exercises (e.g., sleep hygiene, mindfulness); (2) messaging with providers who have partial access to toolbox content; and (3) a peer support forum.
**Green, 2008**	**e-BP**: A website that enables members to communicate with their providers through secured messaging; schedule appointments; refill prescriptions that are then mailed; and view most of the medical record including lab results. In addition to the website, the PHT includes a home blood pressure monitor and direct care supervision from a clinical pharmacist.
**Greiner, 2015**	**MSdialog**: A web- and mobile-based software application that combines information from a handheld electronic autoinjector for subcutaneous drug administration with real-time data regarding administration, clinical outcomes, and patient-reported outcome measures recorded by patients.
**Guo, 2019**	The PHT consists of three digital devices: (1) a cloud-based, tablet computer–accessed, remote monitoring service platform that collects and integrates patients' data from the clinic, hospital information system, and patients; (2) a personal web-based health tracking mobile app for patients; and (3) a few smart health tracking devices.
**Haas, 2019**	**Oviva App**: A mobile app that connects patients remotely with their dietitian and includes the following features: chat-like communication with dietitians; group chat with peers; personal profile; photo-based food log; activity and weight logs; goal scorecard; content database; feedback; and links to educational materials.
**Haze, 2013**	A smartphone application, featuring a secure web-based design with dashboard including health assessment; personalized health plan; disease-specific education; and communication for follow-up evaluation.
**Jelin, 2012**	A website accessed by smartphone featuring an electronic diary for registration of activity, pain, emotions, catastrophizing thoughts; and situational feedback from nurse-therapist.
**Levine, 2009**	**MyCareTeam**: A web-based disease management system that enables patients to transmit blood sugar readings from standard glucose meters; communicate with their healthcare professionals; and access educational material.
**Lie, 2019**	**eGSD**: A web portal for self-management support with the following features: reflection sheets focusing on patients' experiences of living with diabetes; focus for change; mutual planning changes and problem solving; strategies for conducting changes in daily life; and feedback by nurses.
**Lv, 2017**	**EMPOWER-H**: A web-based disease management system that includes: a wireless blood pressure monitor that transmits home blood pressure readings to the electronic health record and the EMPOWER system; a smartphone with 2 apps for transmission of home blood pressure data and for displaying patient-generated data, visualization and tracking of personal goals, and access to educational nuggets; a comprehensive dashboard of the patient's personalized action plan, treatment goals, and self-monitoring data; a pedometer; a web-based messaging system for communicating between patients and the care team; consultation by healthcare staff; and patient-specific text and video educational nuggets.
**Miller, 2011**	**MCCO**: An internet-based system of asynchronous electronic messaging between clinicians and patients. Features: self-monitoring of symptoms; graphical feedback; email prompts; contact with clinical team; links to educational material; and appointment preparation.
**Opipari-Arrigan, 2020**	**Orchestra**: A patient- or parent-facing mobile app and linked to a clinician-facing web-based dashboard with the following features: real-time sharing and visualization of clinical and patient-generated data (symptoms and general health); automated symptom surveillance; actionable alerts; pre-visit health report; and collaborative pre-visit planning.

**Petersen, 2019**	**ImagineCare**: A delivery model consisting of: (1) a 24/7 care support center with staff trained in behavioral change; (2) a clinical workflow application; (3) a mobile app with companion Bluetooth-enabled devices for the participants; and (4) a cloud-based data processing solution. The iOS-developed app had 3 core functional areas: health data, personal profile, and secure messaging.
**Pinsker, 2008**	A therapy-management system with three main components: a health data center that stores information about home measurements of vital parameters, intake of medication or other health parameters provided by patients, and medical examinations provided by caregivers; a PC-based caregiver-terminal with a web portal to access patients' health data and to register data; a mobile-based patient-terminal to enable patients to record and transmit data, get automatically generated reminders and text-based feedback messages.
**Ralston, 2009**	A web-based program for reviewing online medical records, sending blood glucose readings, and sending secure emails.
**Ruland, 2013**	**WebChoice**: An Internet-based, interactive health communication application with the following features: patient self-monitoring of symptoms and problems; individually tailored information and self-management support; e-communication with expert cancer nurses; and an e-forum for group discussion with other patients.
**Tang, 2013**	**EMPOWER-D**: A web-based disease management system that includes: (1) wireless glucometer upload system that transmits home glucometer readings to the electronic health record; (2) diabetes summary status report consisting of a comprehensive, patient-specific 'dashboard' of the status of a patient's personalized action plan and treatment goals, diabetes complications risk, monitoring tests, medications, and health maintenance schedule; (3) nutrition log; (4) insulin record; (5) exercise log; (6) online messaging system for communicating with members of the patient's healthcare team; (7) consultation by healthcare staff; (8) patient-specific text and video educational 'nuggets'.
**Triantafyllidis, 2015**	**SUPPORT-HF**: The system consists of a patient-facing tablet computer, sensing devices (blood pressure monitor, weighting scales, pulse oximeter), and a back-end web-based application for clinicians. The patient interface contains symptom-specific questionnaires; a self-measurement component; educational material (e.g., videos and documents); graphical displays; communication with clinicians. The clinician interface contains graphical displays of patient data; patient message service; activation/deactivation of the self-monitoring component for patients; patient data input; review and assess patient interactions with the system.
**Voruganti, 2017**	**Loop**: A secure online communication tool for team-based clinical collaboration that enables patients and caregivers to communicate asynchronously with multiple members of the health care team involved in providing their direct care, as well as for health care providers including physicians, nurses, and allied health professionals to communicate with each other.
**Zhang, 2019**	**Welltang**: A smartphone-based diabetes management platform that contains education; self-management (including real-time uploading of self-monitored blood glucose, diet, exercise, medication, body weight and other diabetes data); patient community; and communication between patients and clinicians.

Charted variables of included papers

First author, publication year	Country	Journal	Chronic condition (classification)	Levels of care	Study Aim	Study Design	Sample Size	Evaluation results
**Andersen, 2011**	Denmark	Int J Med Inform	Chronic heart patients with ICDs (cardiovascular)	Primary, secondary & tertiary care & other	Design and implementation	Qualitative	<50	Improvement

**Barenfeld, 2020**	Sweden	PLoS one	Chronic obstructive pulmonary disease or chronic heart failure (cardiovascular and pulmonary)	Primary care	User experiences	Qualitative	<50	Improvement
**Bjerkan, 2014**	Norway	J Multidiscip Healthc	Adults with severe psychiatric diagnoses and children with various disabilities (psychiatric disorders)	Primary & secondary care & other	User experiences	Qualitative	<50	Mixed results
**Boon, 2020**	Spain	J Cyst Fibros	Cystic fibrosis (genetic disorders)	Secondary & tertiary care	Effects	Quantitative	151-200	Improvement
**Børøsund, 2014**	Norway	J Med Internet Res	Breast cancer (cancer)	Secondary care	Effects	RCT	151-200	Improvement
**Canan, 2020**	USA	PLoS one	HIV (immunodeficienc y)	Tertiary care	Effects	Quantitative	101-150	Improvement
**Cho, 2009**	South Korea	J Telemed Telecare	Yype 2 diabetes (metabolic disorders)	Secondary care	Effects	RCT	51-100	Improvement
**de Jong, 2017**	Netherlands	Inflamm Bowel Dis	Inflammatory bowel disease (inflammatory disease)	Secondary & tertiary care	Feasibility	Quantitative	<50	Improvement
**Fiks, 2015**	USA	Pediatrics	Pediatric asthma (pulmonary)	Primary care	Feasibility	RCT	51-100	Improvement
**Fishman, 2013**	USA	Am J Manag Care	Hypertension (cardiovascular)	Primary care	Effects	RCT	>200	Improvement

**Flickinger, 2020**	USA	Telemed J E Health	HIV (immunodeficiency)	Primary & secondary care	Design and implementation	Qualitative	151-200	N/A
**Floch, 2020**	Norway	JMIR Mhealth Uhealth	Cystic fibrosis (genetic disorders)	Secondary & tertiary care	Effects	Mixed methods	151-200	Improvement
**Ford, 2019**	USA	Telemed J E Health	Psoriasis (autoimmune disorders/skin disorders)	Primary & secondary care	Effects	RCT	>200	Improvement
**Gall, 2020**	Germany	Int J Med Inform	Dementia (neurodegenerative disorder)	Primary care & other	User experiences	Qualitative	<50	Improvement
**Gammon, 2017**	Norway	J Med Internet Res	Mental health problems (psychiatric disorders)	Primary & secondary care	User experiences	Mixed methods	51-100	Mixed results
**Green, 2008**	USA	JAMA	Hypertension (cardiovascular)	Primary care	Effects	RCT	>200	Improvement
**Greiner, 2015**	USA	Patient	Multiple sclerosis (autoimmune neurological disorder)	Secondary care	User experiences	Mixed methods	>200	Improvement
**Guo, 2019**	China	JMIR Mhealth Uhealth	Chronic heart failure (cardiovascular)	Secondary care	Feasibility	Mixed methods	51-100	Improvement
**Haas, 2019**	Switzerland	JMIR Mhealth Uhealth	Obesitas (metabolic disorders)	Primary & secondary care	Feasibility	Quantitative	<50	Improvement
**Haze, 2013**	USA	Comput Inform Nurs	Asthma (pulmonary)	Primary care	Feasibility	Mixed methods	<50	Improvement
**Jelin, 2012**	Norway	Pain Manag Nurs	Fibromyalgia (chronic conditions, unspecified)	Primary care	User experiences	Qualitative	<50	Mixed results

**Levine, 2009**	USA	J Diabetes Sci Technol	Type 1 and type 2 diabetes (metabolic disorders)	Primary care	Feasibility	Quantitative	101-150	Improvement
**Lie, 2019**	Norway	Scand J Caring Sci	Type 2 diabetes (metabolic disorders)	Primary care	User experiences	Qualitative	<50	Mixed results
**Lv, 2017**	USA	J Med Internet Res	Hypertension (cardiovascular)	Primary care	Effects	Quantitative	101-150	Improvement
**Miller, 2011**	USA	Telemed J E Health	Multiple Sclerosis (autoimmune neurological disorder)	Secondary & tertiary care	Effects	RCT	151-200	No change
**Opipari-Arrigan, 2020**	USA	JMIR Mhealth Uhealth	Inflammatory bowel disease and cystic fibrosis (genetic disorders and inflammatory)	Secondary care	Feasibility	Quantitative	51-100	Improvement
**Petersen, 2019**	USA	JMIR Mhealth Uhealth	Chronic conditions, unspecified	Primary & secondary care	Effects	Quantitative	>200	No change
**Pinsker, 2008**	Austria	Lecture Notes in Computer Science	Type 2 diabetes (metabolic disorders)	Tertiary care	Design and implementation	Quantitative	<50	Improvement
**Ralston, 2009**	USA	Diabetes Care	Type 2 diabetes (metabolic disorders)	Primary care	Effects	RCT	51-100	No change
**Ruland, 2013**	Norway	Cancer Nurs	Breast and prostate cancer (cancer)	Secondary care	Effects	RCT	>200	Slight improvement
**Tang, 2013**	USA	J Am Med Inform Assoc	Type 2 diabetes (metabolic disorders)	Primary care	Effects	RCT	>200	Slight improvement

**Triantafyllidis, 2015**	UK	Int J Med Inform	Heart failure (cardiovascular)	Secondary care	Design and implementation	Mixed methods	<50	N/A
**Voruganti, 2017**	Canada	J Med Internet Res	Stage III-IV cancer (cancer)	Secondary care	Feasibility	RCT	51-100	Mixed results
**Zhang, 2019**	China	J Med Internet Res	Type 1 or type 2 diabetes (metabolic disorders)	Tertiary care	Effects	RCT	>200	Improvement

N/A: Not applicable
